# Efficacy and Safety of Miltefosine in Treatment of Post-Kala-Azar Dermal Leishmaniasis

**DOI:** 10.1155/2015/414378

**Published:** 2015-01-01

**Authors:** Shyam Sundar, Anup Singh, Jaya Chakravarty, Madhukar Rai

**Affiliations:** ^1^Department of Medicine, Institute of Medical Sciences, Banaras Hindu University, Varanasi, Uttar Pradesh 221005, India; ^2^Department of Medicine, All India Institute of Medical Sciences, New Delhi 110029, India

## Abstract

*Background*. Long regimens for the treatment of post-kala-azar dermal leishmaniasis (PKDL) result in noncompliance. A safe, effective, and acceptable regimen for the treatment of PKDL is still to be developed. Miltefosine has been found to be effective in the treatment of Visceral Leishmaniasis (VL). Hence, its efficacy was tested in patients of PKDL. *Methods*. In this exploratory study, 33 patients with PKDL aged 10 years and above were administered miltefosine (50 mg for those weighing <25 kg or 100 mg in divided doses for those ≥25 kg and 2.5 mg per kg for children) for 12 weeks and followed up for one year to find out the efficacy. *Results*. Out of 33 patients, 3 patients withdrew consent. Treatment was stopped due to adverse effect in 1 patient. 28 (96.6%) got cured with complete disappearance of lesion while 1 patient (3.4%) failed treatment by protocol analysis. *Conclusion*. Miltefosine was found to be effective and safe in the treatment of PKDL.

## 1. Introduction

Post-kala-azar dermal leishmaniasis (PKDL) cases serve as a reservoir of* Leishmania *infection in the population and should be diagnosed and treated effectively. PKDL is characterized by macular, papular, or nodular lesions or a mixture of them (Figures 2 and 4). It is quite common in Sudan occurring in >50% patients with Visceral Leishmaniasis (VL), where it may occur concurrently with or immediately following an episode of VL and healing spontaneously in 6 months to several years after an episode of VL, whereas in India it occurs 6 months to 3 years after the cure of VL in <10% of VL cases [1]. Miltefosine has shown its efficacy in the treatment of VL and was recommended as the first-line therapy by WHO for the treatment of VL in Indian subcontinent [2, 3]. Also of concern is the safety profile of miltefosine because for PKDL miltefosine is given for much longer duration.

This trial evaluates the safety and efficacy of miltefosine in cases of PKDL in Bihar state.

## 2. Material and Methodology

An exploratory study was carried out at the Kala-Azar Medical Research Center, Muzaffarpur, at the field site of the Institute of Medical Sciences, Banaras Hindu University. PKDL patients were enrolled between July 2009 and June 2010 and followed up for one year after treatment. The study was approved by the institutional ethics committee, and a written informed consent was taken from each enrolled patient and from the parents of the patient <18 years.

Patients of both sexes, aged more than 10 years, with skin lesions consistent with PKDL (nodules, papules, plaques, or macules) identified by a qualified and trained doctor at Kala-Azar Medical Research Center, Muzaffarpur, Bihar, with or without history of an episode of VL in the past were included in the study. The immunochromatographic rK39 strip test (Inbioss, USA) was performed on serum of all the patients. Demonstration of* Leishmania* infection was done by visualizing amastigotes in slit-skin smear of all patients. However, taking into account the decreased slit-skin smear parasite and culture positivity in diagnosis of PKDL we did only polymerase chain reaction (PCR) for making the diagnosis [4]. DNA isolation was done by resuspending the slit-skin sample in 200 *μ*L phosphate-buffered saline (PBS) and using QIAamp Blood DNA Mini Kit (Qiagen) according to the manufacturer's instructions. Parasite detection by PCR was done by using* L. donovani* species specific primers and methodology was followed as described elsewhere [5]. PCR and k39 positive confirmed cases were only included in the study. However for followup and treatment efficacy only clinical parameters were used.

Exclusion criteria were laboratory biochemical abnormalities (platelet count <100 × 10^9^/L, leukocyte count <2.5 × 10^9^/L, hemoglobin <8.0 g/100 mL, liver function tests ≥3 times the upper limit of normal range, bilirubin ≥2 times the upper limit of normal range, and serum creatinine or blood urea nitrogen ≥1.5 times the upper limit of normal range); major surgery within the last 2 weeks; any noncompensated or uncontrolled condition, such as active tuberculosis, malignant disease, severe malaria, HIV, or other major infectious diseases; lactation, pregnancy, or likelihood of inadequate contraception in females of childbearing potential for the treatment period plus 2 months thereafter; treatment with any antileishmanial drug within the previous 12 weeks.

### 2.1. Drug Treatment

Miltefosine was administered at a target dose of 2.5 mg/kg/day for 12 weeks. Patients ≥25 kg received 100 mg per day: one 50 mg capsule in the morning and evening with meals. Patients <25 kg received 50 mg/day: one 50 mg capsule per day. The patients were treated as inpatients for the first 4 weeks and then continued as outpatients for the rest of the treatment period.

For monitoring the adverse events, except nephrotoxicity, common toxicity criteria of the National Cancer Institute were used [6]. If there was toxicity of grade 3 and above, the treatment was discontinued and the subject was removed from the study and offered rescue treatment. Nephrotoxicity was defined as an increase in serum creatinine that was either double the baseline levels or more than 2.0 mg per deciliter (177 *μ*mol per liter).

### 2.2. Assessment of Efficacy

Efficacy was assessed by decrease in size or disappearance of the lesion. Cure was defined as complete disappearance of skin lesion(s) after treatment, as reported by the patient and assessed by the trained physician at 12-month followup.

### 2.3. Rescue Treatment

Those patients who failed treatment in the form of no response increase in number and size of lesion at 1-year followup and those patients in whom treatment was stopped due to adverse events were offered rescue treatment with three 20-day courses of amphotericin B in doses of 1 mg/kg given 20 days apart [7].

### 2.4. Statistical Analysis

The data were analyzed by using SPSS-16 Version. The data were checked for assumption of normality. Comparison of means was done by using Wilcoxon signed-rank test for paired data. A *P* value less than 0.05 (<0.05) was considered as statistically significant.

## 3. Results

Thirty-three patients with PKDL were included in the study out of which two patients had no history of prior episode of VL. The baseline and follow-up biochemical characteristics of patients are shown in Table 1. As per weight, 5 patients received miltefosine 50 mg once daily and the rest received 50 mg twice daily for 12 weeks.

Amongst the previously treated patients for VL, 18 patients were treated with sodium stibogluconate (SSG), 10 patients were treated with paromomycin, and 1 patient was treated with each of miltefosine, liposomal amphotericin B, and amphotericin B. The median duration of interval between treatment of VL and development of PKDL was 24 months (range: 4 months to 15 years).

Among the various forms of skin lesions 14 patients had exclusively hypopigmented macular patches. Five patients had exclusively nodular lesion and 14 patients had both nodular and macular lesions (Figure 3). PCR was positive in all the cases. However, only 13 patients were parasite positive in split smear.

### 3.1. Safety

In one patient treatment was stopped due to repeated vomiting and diarrhea (CTC grade 4). Three patients withdrew the consent and did not complete the treatment.

### 3.2. Efficacy

**Figure 1:**
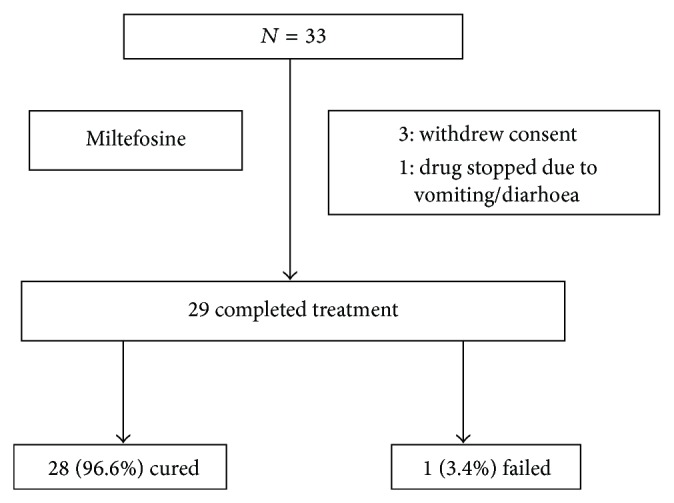
Disposition of patients.

**Figure 2:**
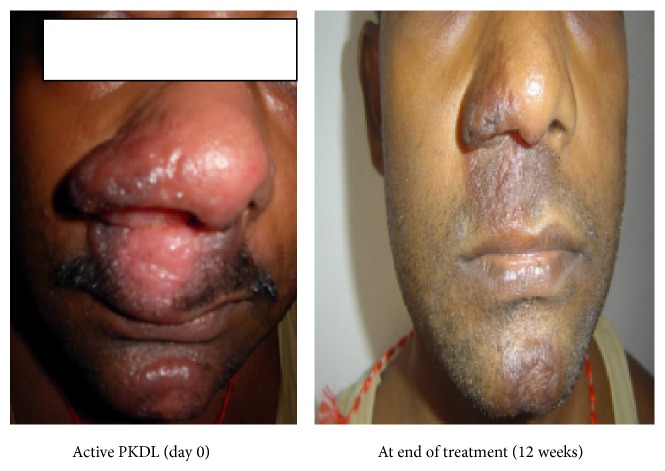
Case 1: patient with nodular lesions.

**Figure 3:**
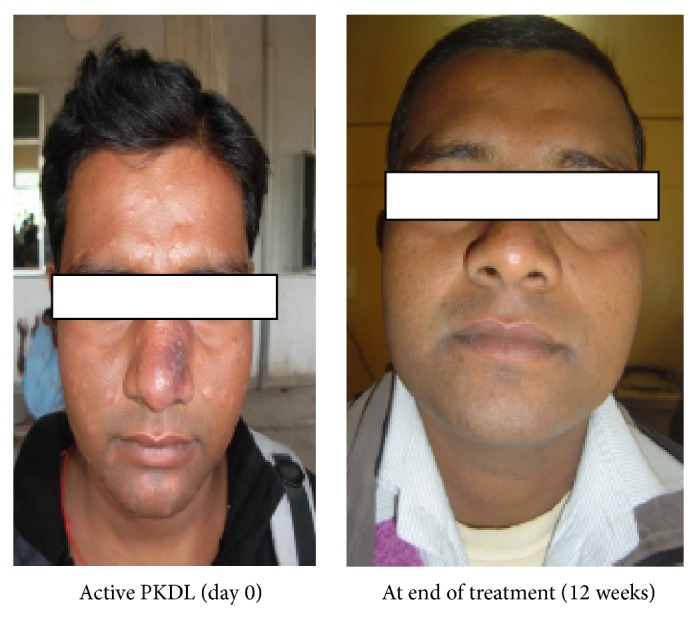
Case 2: patient with both nodular and macular lesions.

**Figure 4:**
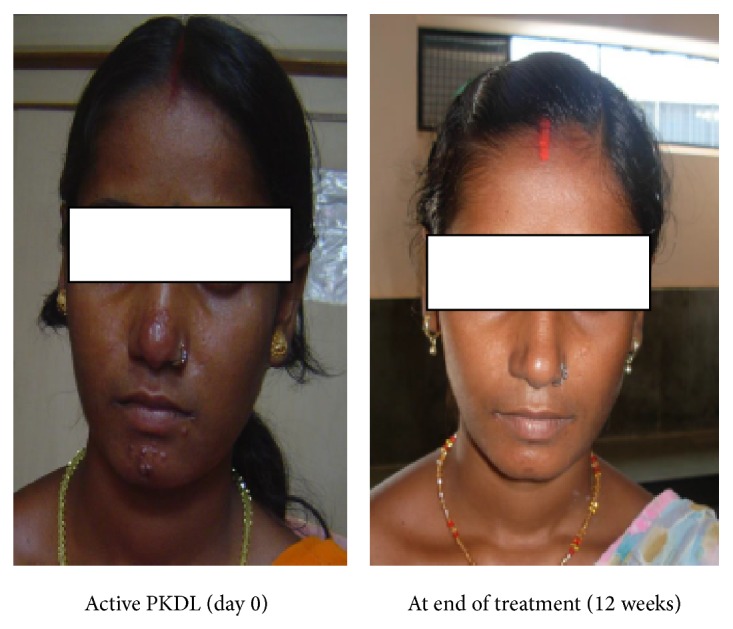
Case 3: patient with nodular lesions.

At one-year followup, out of 29 patients, 28 (96.6%) were cured with complete disappearance of lesion while 1 (3.4%) patient who had nodular lesion before the start of therapy with parasitological score 3+ on split smear examination showed no improvement (Figure 1).

## 4. Discussion

PKDL cases act as major reservoir of infection and hence it is important to identify cases early and treat them accordingly. VL elimination programme run by three countries aims to eliminate VL by 2015 and PKDL in the subcontinent by 2018 [8]. Treatment of PKDL is an unresolved issue. Clinical response may differ according to the type of lesions in PKDL; nodules and papules disappear in 120 days and macules in 200 days [9]. Thus it becomes imperative to follow these patients for a sufficiently long duration to see the long-term effects of the treatment.

Among the various drugs used antimony injections daily for 120 days though effective are associated with side effects like arthralgia and myalgia, pancreatitis, transaminitis, headache, hematologic suppression, and rash [9]. Amp B given as three courses of 20 daily infusions with an interval of 20 days in between the courses is also recommended but the major limiting factors are the side effects associated with it like fever, chills, renal toxicity, and possible cardiorespiratory toxicity [7].

Miltefosine has been tried in different doses and duration for the treatment of PKDL. In Bangladesh, there is a report about the efficacy of miltefosine at a higher dose of 50 mg TID for 60 days (with a need to extend to 90 days if required) in a small number of PKDL patients [10]. The major concern is the gastrointestinal side effect which leads to noncompliance with the drug. Therefore, the therapy should always be directly observed as in our study. Limitation of the study includes the low sample size which could be minimized by involving other recruitment centres in future studies.

Our study showed excellent efficacy of miltefosine (96.6%). Also, tolerability of miltefosine was good except for one patient who had gastrointestinal side effect in the form of repeated vomiting. Recent trails on miltefosine used in the treatment of VL have shown decrease in its efficacy which has raised the concern for its use as a monotherapy for the treatment of PKDL because of the more chances of the emergence of resistance [11]. Therefore, combination therapy should be used as in case of VL to shorten the duration of treatment and thus decrease the chances of drug resistance.

## Figures and Tables

**Table 1:** Hematological and biochemical characteristics of patients at baseline and at 2, 4, and 12 weeks.

	0 week	2 weeks	4 weeks	12 weeks	*P* value		
					0 versus 2	0 versus 4	0 versus 12
WBC(/*µ*L) Median (IQR)	8500.00 (6950.00–10600.00)	9200.00 (7250.00–10300.00)	9300.00 (7850.00–11000.00)	8100.00 (6200.00–10900.00)	0.029	0.138	0.808
Hemoglobin (gm/dL) Median (IQR)	11.600 (10.100–13.850)	11.400 (10.500–13.000)	11.400 (9.900–12.950)	11.000 (8.650–12.950)	0.500	0.715	0.070
Platelets (/*µ*L) Median (IQR)	252000 (216000–309000)	278000 (224000–354000)	302000 (251500–374000)	278000 (169000–338000)	0.013	0.009	0.955
BUN (mg/dL) Median (IQR)	9.40 (7.17–12.08)	8.93 (6.28–10.68)	10.30 (8.58–11.58)	9.635 (7.050–9.635)	0.306	0.330	0.961
SGOT (IU) Median (IQR)	27.00 (19.50–32.50)	26.00 (20.50–30.50)	24.00 (10.50–30.50)	27.00 (16.50–36.00)	0.617	0.206	0.851
SGPT (IU) Median (IQR)	31.00 (23.00–37.00)	28.00 (21.00–39.00)	29.00 (19.50–48.50)	23.00 (14.50–34.00)	0.631	0.432	0.033
Serum creatinine (mg/dL) Median (IQR)	0.6800 (0.5800–0.8800)	0.7500 (0.6600–0.9250)	0.780 (0.650–1.045)	0.7400 (0.5850–1.0850)	0.006	0.004	0.194

IQR: interquartile range.

BUN: blood urea nitrogen.

SGOT: serum glutamic oxaloacetic transaminase.

SGPT: serum glutamic pyruvic transaminase.
